# Pulsed Field Ablation for Atrial Fibrillation in Fontan Palliation

**DOI:** 10.1111/jce.16795

**Published:** 2025-07-09

**Authors:** Fouad Khalil, Abhishek J. Deshmukh, Shane T. Tsai

**Affiliations:** ^1^ Department of Cardiovascular Medicine University of Nebraska Medical Center Omaha Nebraska USA; ^2^ Department of Cardiovascular Medicine Mayo Clinic Rochester Minnesota USA

**Keywords:** atrial fibrillation, catheter ablation, Fontan palliation, pulmonary vein isolation, pulsed‐field ablation

## Abstract

**Introduction:**

Pulsed‐field ablation (PFA) is a novel and effective modality for ablation in patients with atrial fibrillation (AF). Available case reports and small series showed promising results in patients with congenital heart disease. However, PFA ablation in Fontan patients has not been reported yet.

**Methods and Results:**

We present a 35‐year‐old patient with a history of Fontan palliation for hypoplastic left heart who underwent successful ablation for AF with PFA. We describe procedure characteristics and illustrate the potential benefits of PFA in this patient population.

**Conclusion:**

We present a case of PFA for AF in Fontan palliation. PFA was feasible and successful in our case. Long‐term outcomes are yet to be determined.

**Abbreviations**
ACHD: adult congenital heart disease
ACT: activated clotting time
AF: atrial fibrillation
PFA: pulsed‐field ablation
PVI: pulmonary vein isolation

## 1 Introduction

Pulsed‐field ablation (PFA) is a novel, non‐thermal ablation modality that utilizes high‐voltage electrical energy to induce cell death [1]. Data on PFA in patients with congenital heart diseases (CHD) are very scarce. We present a case of successful PFA for AF in a patient with Fontan palliation.

## 2 Case Presentation

A 35‐year‐old male presented to the hospital with decompensated heart failure. The patient has a past medical history of hypoplastic left heart syndrome. He was initially managed with Norwood procedure within a few days after birth followed by hemi‐Fontan procedure when he was 7 months old. He underwent a modified lateral tunnel Fontan procedure at the age of 2 years and subsequent fenestration with left pulmonary artery stent at the age of 4 years. At age 19 years, he developed AF and was started on sotalol.

Presently, during hospitalization, the patient was noted to be in AF despite sotalol. Amiodarone was suboptimal due to age, and he was not a candidate for other antiarrhythmic drugs due to decompensated heart failure in addition to liver and kidney dysfunction. Hence, the decision was made to proceed with catheter ablation. The patient spontaneously converted to sinus rhythm before the procedure and remained in sinus rhythm during the procedure. Anticoagulation (dabigatran) was interrupted only for the night before and the morning of the procedure. The procedure was performed under general anesthesia. A decapolar catheter was advanced into the lateral tunnel and positioned for reference atrial signals and pacing. Intracardiac echocardiography was used to delineate Fontan, atria, and pulmonary veins anatomy. Heparin was given with additional boluses to maintain ACT > 350 s.

**Figure 1:**
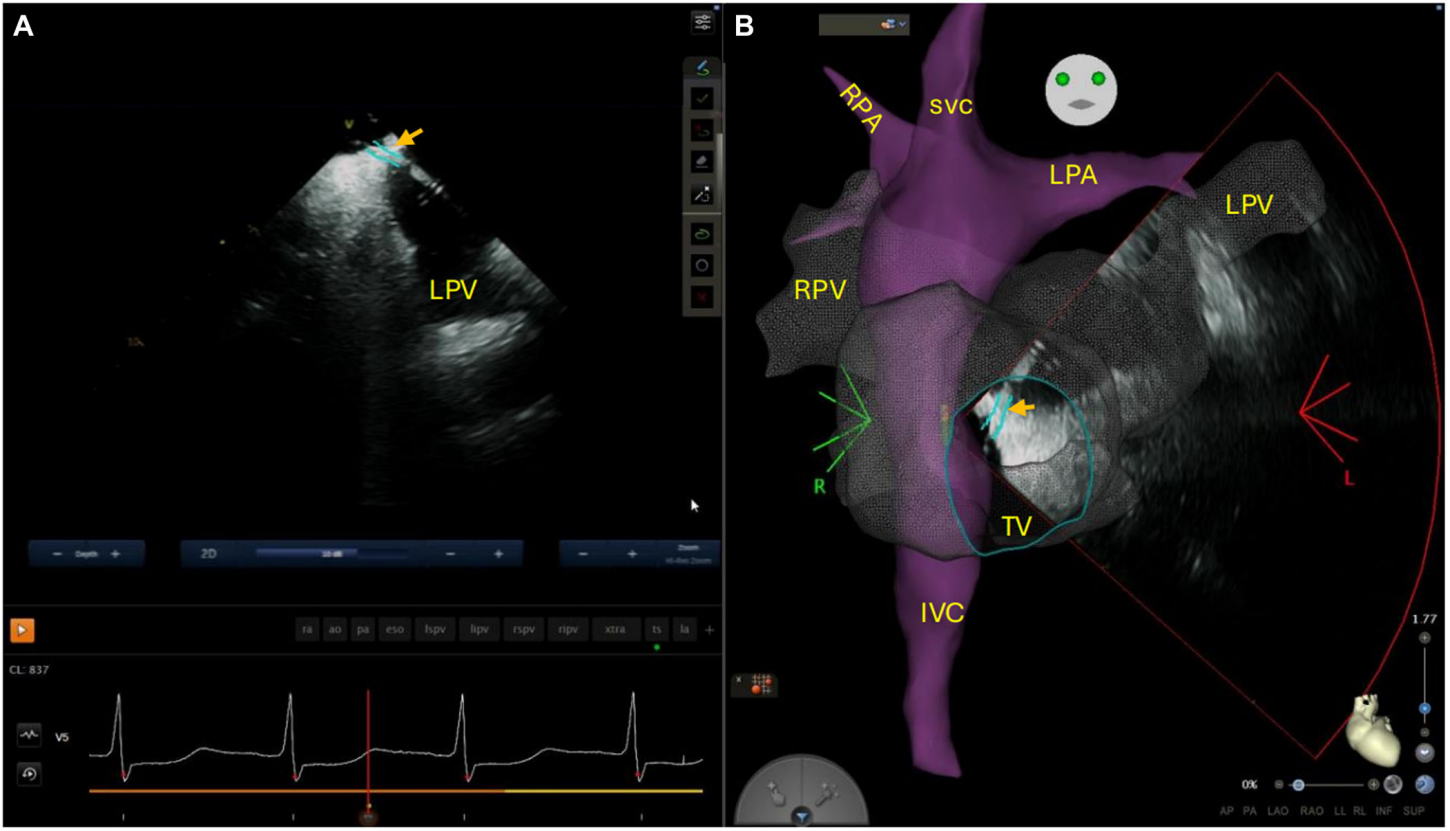
A transbaffle intracardiac echocardiogram (A) showing puncture site (orange arrow) with superimposition of a three‐dimensional electroanatomic map of the lateral tunnel Fontan (B). IVC, inferior vena cava; LPA, left pulmonary artery; LPV, left pulmonary vein; RPA, right pulmonary artery; RPV, right pulmonary vein; SVC, superior vena cava; TV, tricuspid valve.

**Figure 2:**
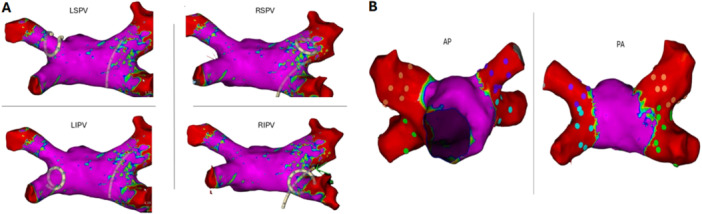
(A) A three‐dimensional electroanatomic map showing individual pulmonary veins isolation with pulsed‐field ablation catheter. (B) Postablation electroanatomic map showing isolation of all four pulmonary veins. LIPV, left inferior pulmonary vein; LSPV, left superior pulmonary vein; RIPV, right inferior pulmonary vein; RSPV, right superior pulmonary vein.

Trans‐baffle access was achieved using AcQCross Qx (Acutus Medical, Carlsbad, CA) system with a target just anterior to the left pulmonary veins and below the superior vein (Figure 1). The needle of AcQCross Qx passed easily through the Fontan baffle. Subsequently, a steerable sheath (VIZIGO, Biosense Webster) was advanced over wire toward the left veins under fluoroscopic and intracardiac echocardiographic visualization with minimal resistance. This access site allowed reasonably good maneuverability and provided adequate reach to both the left and right pulmonary veins. 3D electroanatomic and activation maps of atria and pulmonary veins were performed with an Octaray high‐density catheter using CARTO 3 mapping system (Biosense Webster, Diamond Bar, CA). Low voltage was noted in the lateral tunnel consistent with extensive scar, although relatively normal voltage was noted in the atria. Sheath exchange was then performed over 0.035 F 150 cm Emerald guide wire (Cordis Corporation, Milpitas, CA) for FlexCath Contour (Medtronic, Dublin, Ireland). Pulmonary vein isolation was performed using PulseSelect^TM^ PFA system (Medtronic, Minneapolis, MN, USA). Ostial then antral lesions were applied in a circumferential fashion around the left followed by the right veins (Figure 2). PVI was achieved after a total number of 51 PFA applications. In addition to entrance block, exit block was verified with multi‐site pacing and isolation was confirmed. Programmed atrial stimulation was performed after PFA to verify non‐inducibility of atrial arrhythmias. Total procedure time was 153 min and fluoroscopy time was 5.4 min. Sotalol was continued after the procedure due to frequent premature ventricular contractions. The patient is now 4 months postablation without recurrence.

## 3 Discussion

Although applications of PFA are expanding, data on its use in CHD remain scarce [2, 3]. This report describes a successful case of PFA for recurrent AF in a Fontan patient. While intra‐atrial tachycardia is the most common arrhythmia in Fontan patients, AF is increasingly observed with improved survival in this population [4]. Atrial tachycardia often triggers AF; however, extensive monitoring and intraoperative testing in this case confirmed AF as the primary arrhythmia.

Managing AF in Fontan patients is challenging due to their complex anatomy, necessitating ablation at specialized CHD centers [5]. Challenges with ablation in Fontan patients include accessing arrhythmia substrates, which may require trans‐baffle puncture, as in our case. IVC interruption in heterotaxy patients and venous occlusions can further complicate access, while fragile conduction systems and multiple complex arrhythmias hinder safe and effective ablation [6]. Substrates of AF in Fontan patients are also not well defined. Although PVI remains the cornerstone of AF ablation, successful cases targeting right atrial tissue without PVI have been reported [7, 8].

PFA offers a promising alternative, enabling safe ablation in Fontan patients with larger and transmural lesions in a shorter time. This is crucial given the frequent atrial hypertrophy and extensive scarring in this population. Novel focal PFA catheters may further aid in ablating focal tachycardias that frequently coexist in Fontan patients and often trigger AF [9].

Although no complications occurred in our case, vigilance is required when using PFA near coronary arteries due to the risk of coronary spasms [10]. Multiple reports have demonstrated resolution of PFA‐induced coronary spasms with intracoronary nitroglycerin [3, 10]. Continuous 12‐lead ECG monitoring, alongside availability of urgent coronary angiography and nitroglycerin, is essential. While trans‐baffle access was safely achieved in this case, larger studies are needed to confirm the safety of this approach with PFA sheaths. Additionally, as the experience with PFA is still relatively early, long‐term risks such as fibrosis, arrhythmogenicity, and tissue remodeling remain uncertain.

## 4 Conclusion

We report a case of successful PFA for AF in a patient with Fontan palliation. Our case illustrates the feasibility and effectiveness of PFA ablation in Fontan patients. Future studies are required to confirm PFA safety and durability in this patient population.

## Disclosure

The authors have nothing to report.

## Data Availability

The data that support the findings of this study are available on request from the corresponding author. The data are not publicly available due to privacy or ethical restrictions.
